# Genomic alterations of Tenascin C in highly aggressive prostate cancer: a meta-analysis

**DOI:** 10.18632/genesandcancer.196

**Published:** 2019

**Authors:** Prachi Mishra, Michael A. Kiebish, Jennifer Cullen, Alagarsamy Srinivasan, Aliyah Patterson, Rangaprasad Sarangarajan, Niven R. Narain, Albert Dobi

**Affiliations:** ^1^ Henry Jackson Foundation for Advancement of Military Medicine, Bethesda, MD, USA; ^2^ Center for Prostate Disease Research, USU-Walter Reed Surgery, Bethesda, MD, USA; ^3^ BERG LLC., Framingham, MA, USA; ^4^ Division of Science and Mathematics, University of the District of Columbia, Washington DC, USA

**Keywords:** prostate cancer, neuroendocrine subtype, Tenascin C, TCGA, biomarkers

## Abstract

Tenascin C (TNC), an extra-cellular matrix (ECM) family gene, is expressed in several cancer tissues of breast, lung, colon, and gastrointestinal tract leading to proliferation, migration, invasion, angiogenesis and metastasis, but its role in tumorigenesis of prostate cancer is poorly understood. We took a meta-analysis approach to characterize the alterations of TNC gene in prostate cancer using publicly available databases (cBioportal Version 2.2.0, http://www.cBioportal.org/index.do). The analysis identified TNC alterations (gene amplification) significantly in the neuroendocrine prostate cancer dataset (Trento/Broad/Cornell, *N* = 114), which was further validated in other prostate cancer datasets, including The Cancer Genome Atlas (TCGA) prostate cancer (2015). In the TCGA prostate cancer dataset (*N* = 498), high TNC (alteration frequency, 36%) revealed a strong association with high diagnostic Gleason score. Genomic alterations of TNC was also significantly associated (*P* < 0.05) with expression level of genes from NOTCH, SOX and WNT family, implicating a link between TNC and poorly differentiated aggressive phenotype in NEPC. TCGA prostate adenocarcinoma cases with TNC alteration also demonstrated prominent decrease in disease-free survival (*P* = 0.0637). These findings indicate a possible association of TNC to the aggressive subtype of prostate cancer and warrant further functional studies to evident the involvement of TNC in prostate cancer progression.

## INTRODUCTION

Tenascin C (TNC), one of the four members of tenascin family, is an extracellular matrix (ECM) glycoprotein with a hexameric structure. Each of the monomers, comprise of four domains, a cysteine rich N-terminus TNC assembly (TA) domain, epithelial growth factor EGF-like repeat domain, Fibronectin-type III (FNIII) domain capable of alternative splicing and a calcium rich COOH fibrinogen globe [1] having distinct function and binding affinity for several cell surface receptors, ECM proteins and glycolipids. TNC is a secreted protein, and has major functions in tumor initiation, progression [2] and metastasis [3] [4]. Functionally it facilitates interaction of cells with the ECM and growth factors, and regulates cell adhesion, migration and differentiation [5]. TNC expression is frequently observed in human cancer and increasing evidences show its association with key clinical parameters, such as relapse-free or overall survival. The prognostic value of TNC has been evaluated in non-small cell lung carcinoma, breast and colon cancer [6-8]. Involvement of TNC in the oncogenic process of prostate cancer is evolving.

Biomarkers ranging from RNA (mRNA, lnRNA and miRNA), protein and metabolites have been identified for prostate cancer using various diagnostic approaches. A subpopulation of prostate cancer patients with primary treatments, ultimately develop castration resistant prostate cancer with poor prognosis. Considering the significant role played by extracellular matrix proteins in metastatic cancer, TNC was investigated to understand its role in tumorigenesis or metastasis of prostate cancer in the present study. Few studies have linked TNC to bone metastasis and poor prognosis of prostate cancer [9-11]. In this study, we have utilized a targeted meta-analysis approach based on the information available from published reports and the publicly available data bases to study the role of TNC in sub-populations of prostate cancer at DNA, RNA transcription and protein levels. Here, we identified TNC as a prostate cancer biomarker candidate demonstrating significant alterations in a neuroendocrine dataset, and associated with high diagnostic Gleason score and disease-free survival. These findings indicate the prognostic ability of TNC and its potential contribution to features associated with disease progression.

## RESULTS

### TNC is highly altered in neuroendocrine prostate cancer

**Figure 1:**
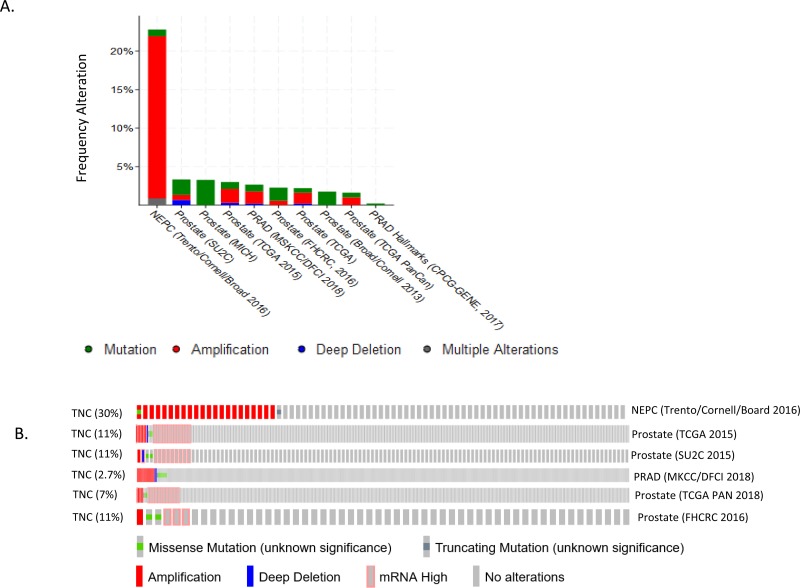
TNC is altered in neuroendocrine prostate cancer tissues **A.** Percentage of alteration frequency of TNC in form of somatic mutation (green), gene amplification (red), deep deletion (blue), multiple alterations (grey) represented across datasets of prostate cancer, present in c-bioportal. **B.** OncoPrint representing the query for TNC across six datasets, as described in the figure. Each row represents oncoprint for the queried gene in a separate prostate cancer dataset. Alteration frequency for the gene is represented in percentage. The rectangular boxes for each dataset oncoprint, represents a patient sample/case. Genetic alterations are color coded: Amplification, red; Gain of function (mRNA High), Pink; Missense mutations, green; Deep deletion, blue; No alterations, grey.

Tenascin C stood out as one of the biomarkers in various cancers [2, 8-10, 12, 13], but its genomic status was unknown in subtypes of prostate cancers. Based on the available literatures, we hypothesized that TNC could be associated with genomic alterations in a subset of prostate cancer tissues. We investigated 18 publicly available prostate cancer datasets through cBioportal (http://www.cBioportal.org/public-portal). Of the 18 datasets, 10 showed significant alteration in the gene (Figure 1A). The datasets which showed TNC alteration included Neuroendocrine prostate cancer (Trento/Broad/Cornell 2016) [14] Metastatic prostate cancer (SU2C/PCF 2015) [15] TCGA 2015[16] The TCGA Pan-Cancer Atlas [17] Prostate Adenocarcinoma MSKCC/DFCI 2018[18] Prostate Adenocarcinoma CRC Nature Medicine 2016 [19]. The alterations in the TNC gene was either in form of gene amplification, mRNA expression, somatic mutation, deep deletion or other alterations. Specifically, the neuroendocrine prostate cancer (NEPC) database showed the most robust alteration (30%, 23 out of 77 sequenced samples in total) in the gene mostly in the form of gene amplification (Figure 1B, Table 1), though small percentage of patients also showed somatic mutations. This observation prompted us to ask whether amplification of TNC is associated with oncogenic driver genes in prostate cancer. We investigated the alteration of other oncogenic drivers (ERG and MYC) in the NEPC and found a significant co-amplification of *TNC* with either *ERG* (27%, *P* < 0.001) or *MYC* (53%, *P* < 0.03) (Supplementary Figure 1, Table 1). Interestingly, there were no instances in which TNC was altered independently of either of the oncogenic drivers. Moreover, all but two cases were amplified for both MYC and TNC, suggesting TNC may have closest association with MYC, in the NEPCs.

**Table 1:** Co-occurrence of TNC with AR, ERG and MYC in NEPC dataset

Gene A	Gene B	Log Odds Ratio	*p*-Value	Adjusted *p*-Value	Tendency
TNC	ERG	>3	<0.001	<0.001	Significant Co-occurrence
TNC	MYC	>3	<0.001	<0.001	Significant Co-occurrence
TNC	AR	1.322	0.005	0.031	Significant Co-occurrence
MYC	AR	1.456	<0.001	0.002	Significant Co-occurrence
ERG	Myc	2.590	<0.001	<0.001	Significant Co-occurrence
ERG	AR	1.329	0.008	0.045	Significant Co-occurrence

### TNC is associated with high Gleason score of prostate cancer

**Figure 2:**
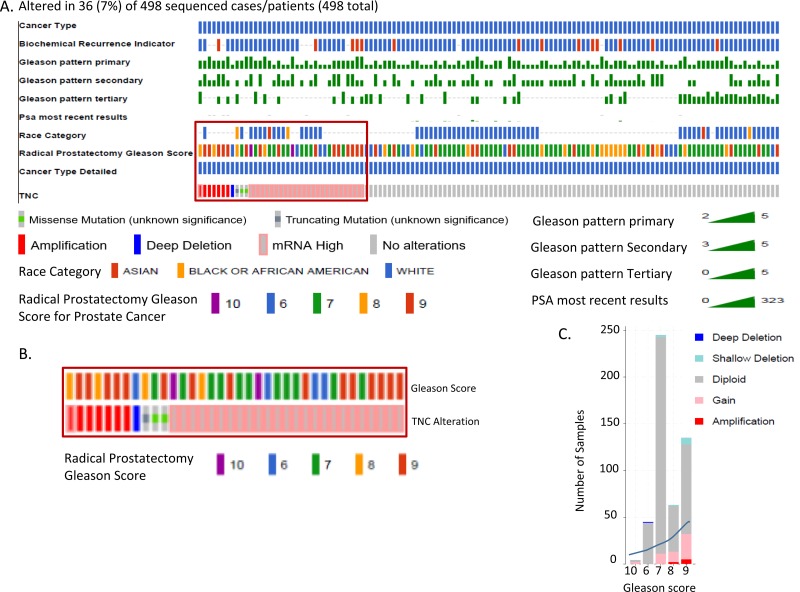
TNC is altered in prostate cancer tissues with high Gleason score (TCGA) **A.** OncoPrint representing alteration of TNC in TCGA dataset with prostate adenocarcinoma cases (Total *N* = 499, *N* = 498 sequenced patient samples). Genetic alterations are color coded: Amplification, red; Gain of function, pink; Missense mutations, green; deep deletion, blue; No alterations, grey. Patient characteristics (color coded separately for each of the characteristics) including Cancer type, Radical prostatectomy Gleason score, Race category and PSA are represented in horizontal rows. **B.** Diagnostic Gleason score in patients with specifically TNC alteration. **C.** Graph showing stratification of TNC altered tumor samples under different Gleason score category. Each bar of the graph represents a class of Gleason score and stratified for the alteration in TNC.

**Figure 3:**
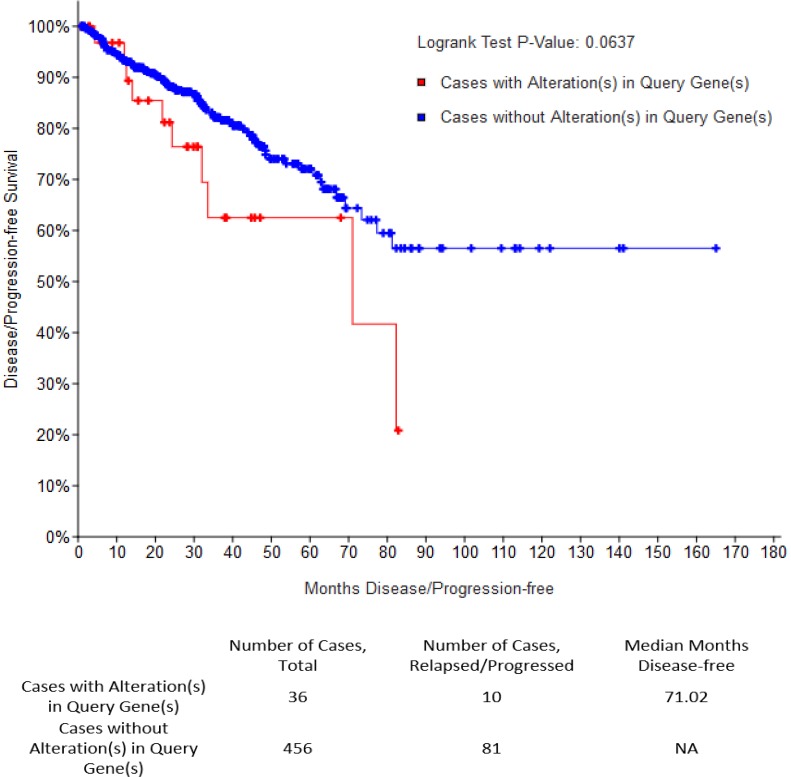
TNC has a possible association with poor disease-free survival (DFS) Kaplan-Meyer curve representing disease free survival (DFS) for total number of cases (*N* = 492) in TCGA prostate adenocarcinoma dataset. Median months of survival distribution for cases with altered TNC (*N* = 36) and unaltered TNC (*N* = 456) were compared using LogRank Test.

In an independent approach, we queried alteration for TNC in TCGA prostate cancer dataset, which revealed that patients with an altered (amplified or overexpressed) TNC, had a Gleason score higher than 7, indicative of future disease progression (Figure 2A, 2B, 2C). Previously TNC had shown an association with disease aggressiveness in breast cancer [3, 8]. Increased copy number and expression of TNC in prostate adenocarcinoma tissues with high Gleason score fortifies the potential link of TNC with disease aggressiveness in prostate cancer as well. This was further supported by the survival report from the TCGA dataset in which patients harboring prostate cancer with TNC alteration showed a trend of decrease in disease-free survival (*P* = 0.0637), although with no difference in the overall survival that may be affected by comorbidity (Figure 3). Our findings of TNC disease-free survival data was consistent with the study performed by Ni et al. in 2017, which reported a poor survival and prognosis of patients with high expression of TNC protein in prostate cancer biopsy tissues, through a histopathological study. We further explored the gene expression data of TCGA, and interestingly found that, unlike the NEPC dataset which demonstrated a co-amplification of *TNC* with *AR, MYC* and *ERG* (Supplementary Figure 1), most of the prostate adenocarcinoma patients in TCGA having TNC alteration, did not show a significant co-amplification of *AR, MYC* and *ERG,* or co-expression of AR and ERG (Supplementary Figure 2). From this we may conclude that, although at genomic levels *TNC, AR, ERG* and *MYC* are co-segregated in some of the neuroendocrine prostate cancers, the expression and function of TNC may not exclusively depend on or linked to AR and ERG.

### TNC is associated with an aggressive phenotype in prostate cancer cells

**Figure 4:**
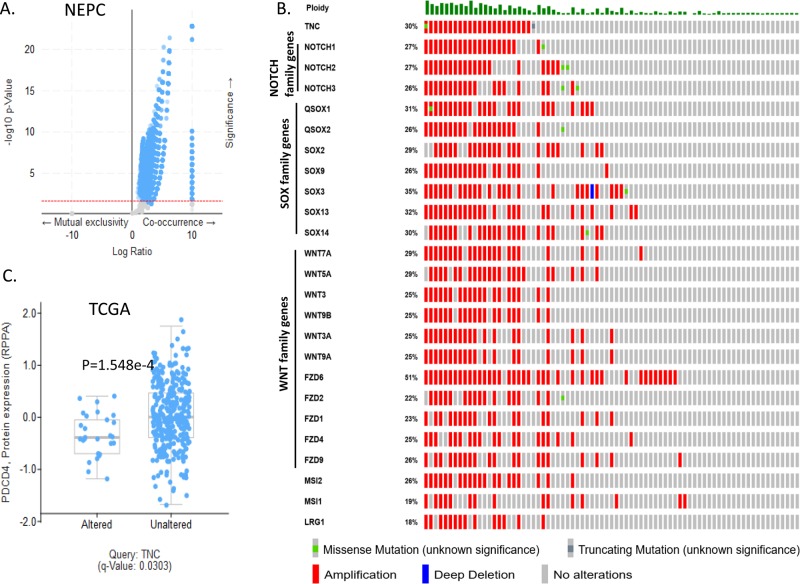
TNC is linked to aggressive tumor promoting phenotype in neuroendocrine prostate cancer cells **A.** Volcano plot showing the number of genes co-amplified (N=17977; putative copy number alterations adjusted by ploidy and purity with CLONET) with TNC in NEPC dataset. X-axis, log10 ratio of positive or negative co-occurrence of the genes with TNC; Y-axis, −log10 P-value. Red dotted line shows the cut of at *P*-value 0.05. **B.** OncoPrint of 24 genes taken for query with TNC for their genomic alteration frequencies in NEPC dataset. Genetic alterations are color coded: Amplification, red; Gain of function, pink; Missense mutations, green; deep deletion, blue; No alterations, grey. Each row of the oncoprint represents the same set of patients for each of the queried gene, alteration frequency for the gene represented in percentage. **C.** Box whisker plot showing protein expression of PDCD4 in TNC altered and unaltered patient population, Significance; *P* < 0.05.

TNC was reported to play an important role in dedifferentiation and metastasis of breast cancer and lung cancer [8, 20]. We hypothesized possible association of TNC with genomic alterations in the genes that contribute to a heterogenous and aggressive phenotype of prostate cancer. Hence, we explored the co-expression, enrichment and network section in both TCGA and NEPC database. We found that in NEPC, *TNC* (cytoband 9q33.1) demonstrated significant co-amplification with the members of NOTCH family (5 genes including *NOTCH1, NOTCH2, NOTCH3*), SOX family (21 genes including *SOX2, SOX3, SOX9, SOX13, SOX14*), WNT family (19 genes including *WNT5A, WNT7A, WNT10B*), FZD family (9 genes including *FZD1, FZD2, FZD4, FZD6, FZD9*), *NANOG, MSI* and *LRG1* (Figure 4A, 4B, Supplementary Table 1). Pearson correlation analysis revealed significant positive correlation of TNC overexpression with expression of *NOTCH1*, *SOX2*, *SOX2 SOX5* and *SOX7* in the TCGA (Supplementary Figure 3). RNA sequencing data demonstrated up-regulation of several genes involved in cell proliferation, de-differentiation and invasion in a significant number of prostate adenocarcinoma patients with high TNC expression (Supplementary Figure 2). Moreover, with TNC copy number increase, protein expression of PDCD4 (programmed cell death 4) was significantly reduced (Figure 4C). PDCD4 plays important role in maintaining the stability of cell proliferation by keeping a normal rate of apoptosis and its decreased expression contributes to disease progression in breast cancer [21].

## DISCUSSION

We identified an enrichment of genomic alteration of TNC in the neuroendocrine subtype of prostate cancers, mainly in the form of gene amplification. TNC demonstrated a stem-cell dominating phenotypic gene signature in independent prostate cancer data sets. In TCGA prostate adenocarcinoma dataset TNC expression or alteration was associated with poor disease-free survival, indicating potential of TNC for being a prognostic biomarker.

TNC is a large secretory glycoprotein which is normally expressed during the process of organogenesis to accompany certain essential process like cell proliferation, migration and epithlial-to-mesenchymal transaction (EMT), but have limited expression in differentiated cells [2]. TNC harbors protein-protein interaction residues for direct binding to other structural proteins like fibronectins and integrins. Increased alterations of TNC from our meta-analysis, in the NEPC dataset indicates the association of TNC with disease aggressiveness in prostate cancer with poor phenotype. Various studies reported that TNC has a significant link to breast, lung, colon, gastric and pancreatic cancer progression with metastatic phenotype [12]. Immunohistochemistry and pathological data have demonstrated an increased expression of TNC in tumor tissues of colon [12] and prostate [10]. Our meta-analysis findings were consistent with these studies as TNC amplification and overexpression was seen in most of the aggressive prostate adenocarcinoma tissues of TCGA with high Gleason score. Moreover, a sharp decline in the status of disease-free survival selectively in the TNC altered subgroup strengthens this finding.

The NEPC dataset of cBioportal had more than 50% of patient with significantly altered oncogenic drivers such as ERG and MYC along with TNC. Strikingly none of the NEPC tumors with TNC amplification was independent of the alterations of the master genes ERG and MYC, indicating a selective co-segregation of TNC along with MYC and ERG amplification in the subtype of aggressive prostate cancers. In primary tumors, ERG is frequently overexpressed in prostate cancer [22] due to genomic fusions, placing ERG under the control of AR regulated promoters [23]. In ERG positive tumors, MYC is activated by ERG [24], suggesting a treacherous path of AR->ERG->MYC axis especially when fixed by genomic amplification. Our result suggests that genomic amplification of AR, ERG and MYC are potential driving forces of the aggressive NEPC with clinical significance, and TNC associates with this treatment resistant subtype.

Our query for the TNC along with other tumor markers revealed a positive correlation for the amplification of TNC with the NOTCH, SOX, WNT and FZD family members, which may partially explain why TNC expression could be related to a poorly differentiated phenotype of the aggressive prostate cancer. The transcription factors SOX2 and SOX9 are potential drivers of invasive prostate cancer that induce EMT and stemness [25] whereas, Wnt signaling promotes tumor microenvironment in progenitor cells in castration resistant prostate cancer (CRPC) enhancing their resistance to therapy [26]. NOTCH1 contributes to proliferation, invasion, oncosphere formation and metastasis in CRPC cells, which can be therapeutically inhibited by gamma secretase inhibitors [27]. Our findings suggest possible interaction of TNC with the potential tumor markers in advanced subtype of prostate cancer, which are also supported by findings from other groups. TNC induced epithelial to mesenchymal transition and proliferation in colorectal cancer [12], which demonstrated its potential to induce metastases. TNC expression in breast cancer animal model, initiated lung metastasis by enhancing WNT and NOTCH signaling and enhancement of stem cell signaling components [8], EMT-like phenotype in colorectal cancer [12] and angiogenesis in lung cancer [20]. Taken together, these findings corroborate the role of TNC in inducing an aggressive and poorly differentiated phenotype. Our results did not rule out the possibility that prostate cancer subtypes with alteration in ERG and MYC may exist in NEPC, without an involvement of TNC through alternative pathways. At present, there are very few studies [28] that link TNC with ERG, but none, that report an association of TNC with MYC. We envision that TNC might be regulated downstream by one or more than one of these oncogenic drivers that in turn regulates the tumor cell growth differentiation. However, in the current study, since majority of the cases in NEPC demonstrated an alteration in the stem cell marker genes associated to TNC and few of them associated exclusively to TNC in TCGA, we conclude that TNC is associated with and potentially contribute to aggressive phenotype, with poor survival and recurrence of advance prostate cancer tissues.

NEPC has extremely poor prognosis with lack of systemic therapies, therefore, a better understanding of molecular alterations in the progression of NEPCs, may improve the therapies. To date, there have been numerous biomarkers identified for poor prognosis of prostate cancer, but very few of them are specific for molecular subtypes. Our study is the first to report an association of TNC with neuroendocrine subtype of prostate cancer in publicly available datasets, further clinical and mechanistic studies are warranted to unravel its functional role in aggressiveness and progression of prostate cancer.

## MATERIALS AND METHODS

### Query for publicly available prostate cancer datasets

We explored the publicly available databases through cBioportal for Cancer Genomics (cBioportal Version 2.2.0, http://www.cBioportal.org/index.do) which contains 18 databases including Neuroendocrine prostate cancer (Multi-Institute 2016, *N* = 114) [14], Metastatic prostate cancer (SU2C/PCF 2015, *N* = 150) [15], The Cancer Genomic Atlas (TCGA, 2015, *N* = 499) [16], The TCGA Pan-Cancer Atlas (*N* = 494) [17], Prostate Adenocarcinoma MSKCC/DFCI 2018 (*N* = 1013) [18], Prostate Adenocarcinoma (FRCRC 2016, *N* = 176) [19], Metastatic prostate adenocarcinoma (SU2C/PCF, *N* = 444) [29], Metastatic prostate adenocarcinoma (MCTP, *N* = 444) [30], Prostate Adenocarcinoma (Broad/Cornell 2013, *N* = 57)[31], Prostate Adenocarcinoma (Broad/Cornell 2012, *N* = 112)[32], Prostate Adenocarcinoma (CPC-GENE 2017, *N* = 477)[33], Prostate Adenocarcinoma (MSKCC 2010, *N* = 216)[34], Prostate Adenocarcinoma (MSKCC 2014, *N* = 104)[35], Prostate Adenocarcinoma (SMMU 2017, *N* = 65)[36], Prostate Adenocarcinoma Organoids (MSKCC 2014, *N* = 12)[37], Prostate Cancer (MSKCC 2017, *N* = 504)[38] and The Metastatic Prostate Cancer Project (provisional 2018). Each of the databases were queried for the status of the TNC gene alteration. Among all The Cancer Genomic Atlas (TCGA) hosted by the Computational Biology Center at Memorial-Sloan-Kettering Cancer Center had prostate cancer dataset with mostly the prostate adenocarcinoma patients (*N* = 499) and the neuroendocrine prostate cancer (NEPC) dataset (*N* = 114) hosted by Trento, Cornell and Broad institute were queried in depth for TNC alteration. In the TCGA prostate cancer, TNC were queried for somatic mutations (obtained by whole exome sequencing, *N* = 498) and copy number alterations on cases (*N* = 492) were determined using GISTIC 2.0., mRNA expression z-scores (RNA seq V2 RSEM) and protein expression Z-scores, measured by reverse-phase protein array (RPPA). Patient characteristics for the TCGA can be found at https://www.cbioportal.org/results/oncoprint. In the NEPC dataset we queried for somatic mutations (obtained by whole exome sequencing, *N* = 77) and copy number alterations (putative copy-number alterations adjusted by ploidy and purity with CLONET, *N* = 77), for a panel of genes of interest, including *TNC*, *AR*, *ERG,* and *MYC*. Patient characteristics for the NEPC was not available in the dataset. Furthermore, a list of 24 stem cell markers were queried for correlation along with TNC amplification in the NEPC, whereas on the other hand, TCGA RNA sequencing data was downloaded and analysis was performed to see the association between expression level of TNC and other genes. OncoPrint was generated after putting the genes into query, to obtain a compact graphical summary of genomic alternations across the tumor samples of the dataset [39]. Each patient or sample were represented as a rectangular box in the OncoPrint. Alteration for each of the queried gene was calculated in terms of percentage of the total number of cases in the OncoPrint. The different types of genomic alterations were represented by different color code. While obtaining OncoPrint from TCGA dataset, patient characteristics were considered.

### Correlation plots, co-expression, network and survival analysis for metadata

Enrichment of other genes with the alteration of TNC (either through copy number alteration, expression of mRNA or protein of other genes), were obtained by the portal and displayed as volcano plots (for all the genes) or box and whisker plots (for individual gene) with −log10 *P*-value and log ratio for their fold change specified for co-occurrence and mutual exclusivity. Co-expression, network and survival analysis were demonstrated as described in Gao et.al., 2013[39]. For overall survival and disease-free survival analysis cases with alteration in TNC (*N* = 36) were compared with cases unaltered for TNC (*N* = 456), and log-rank test was performed.

### Statistical analysis

Statistical analyses were performed by the existing statistical platform in the cBioportal. *P-*value less than or equal to 0.05 was considered significant. Statistics for mutual exclusivity or co-occurrence for each pair of queried gene generated by the portal provided the odds ratio (OR) and *P*-value (derived from Fisher's exact test).

## SUPPLEMENTARY FIGURES AND TABLES



## Abbreviations

TNC: Tenascin C
ECM: Extra Cellular Matrix
NEPC: Neuroendocrine Prostate Cancer
TCGA: The Cancer Genomic Atlas
PDCD4: programmed cell death 4
