# Supplementary material for: Genomic alterations of Tenascin C in highly aggressive prostate cancer: a meta-analysis
Source: Genes Cancer. 2019;10(5-6):150–9. doi: 10.18632/genesandcancer.196 (PMC6872669; doi:10.18632/genesandcancer.196)
Supplement: Supplementary file 1 [file ganc-10-150-s001.pdf]

Genomic alterations of Tenascin C in highly aggressive prostate cancer: a meta-analysis – Mishra et al

Supplementary Figures

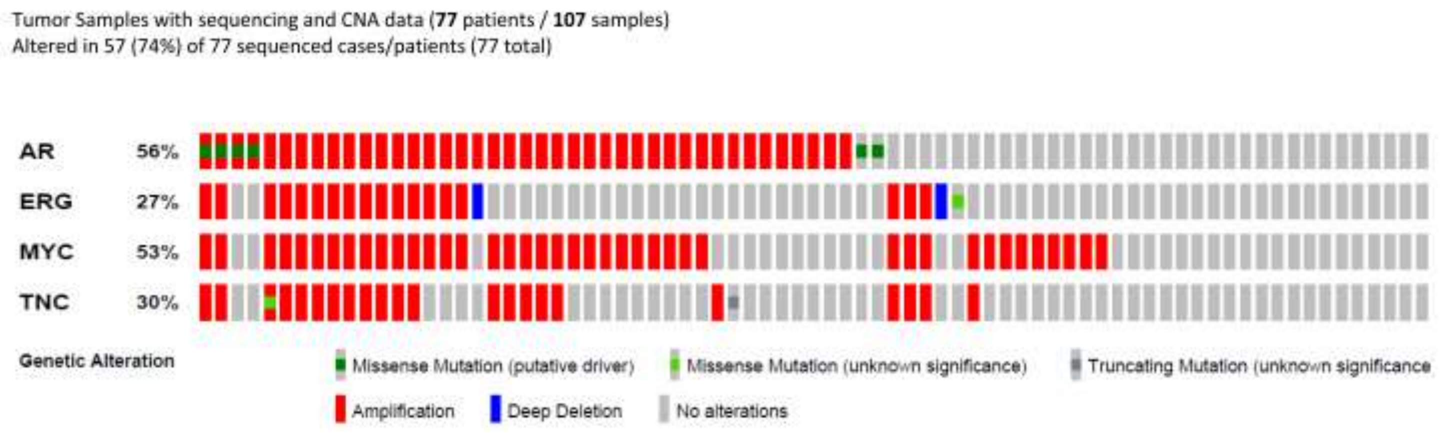

Supplementary Figure 1: TNC alterations in NEPC. OncoPrint representing the query for TNC along with AR, ERG and MYC among the neuroendocrine prostate cancer dataset (*N* = 104). Number of samples with sequencing and CAN data were *N* = 77. Each row of the oncoprint represents the same set of patients for each of the queried gene, alteration frequency for the gene represented in percentage. Each rectangle box in the figure represents a patient sample/case. Genetic alterations are color coded: Amplification, red; Missense mutations, green; deep deletion, blue; No alterations, grey.

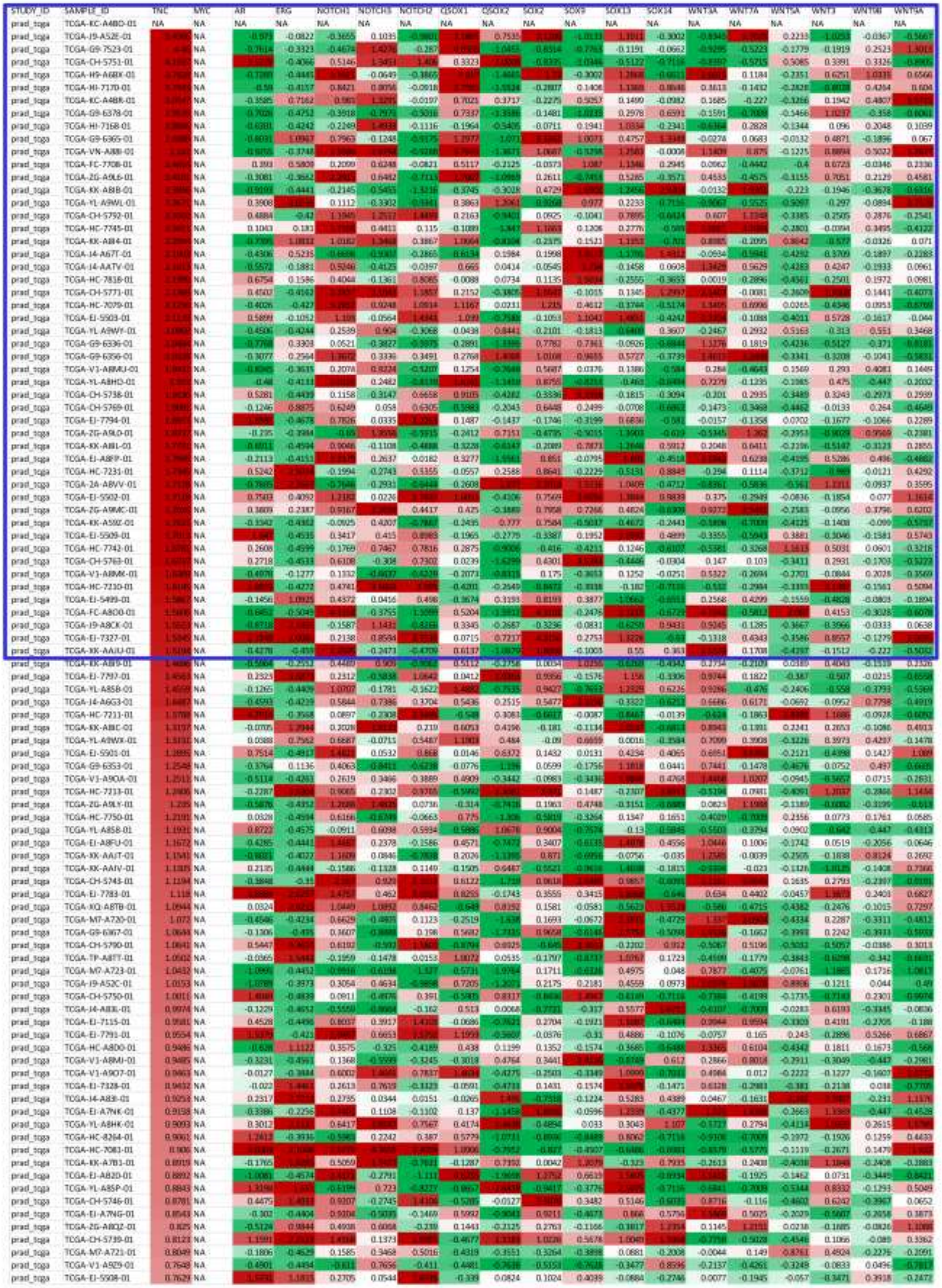

Supplementary Figure 2: mRNA expression correlated to TNC gene alterations in TCGA (top 100 cases with TNC alteration)

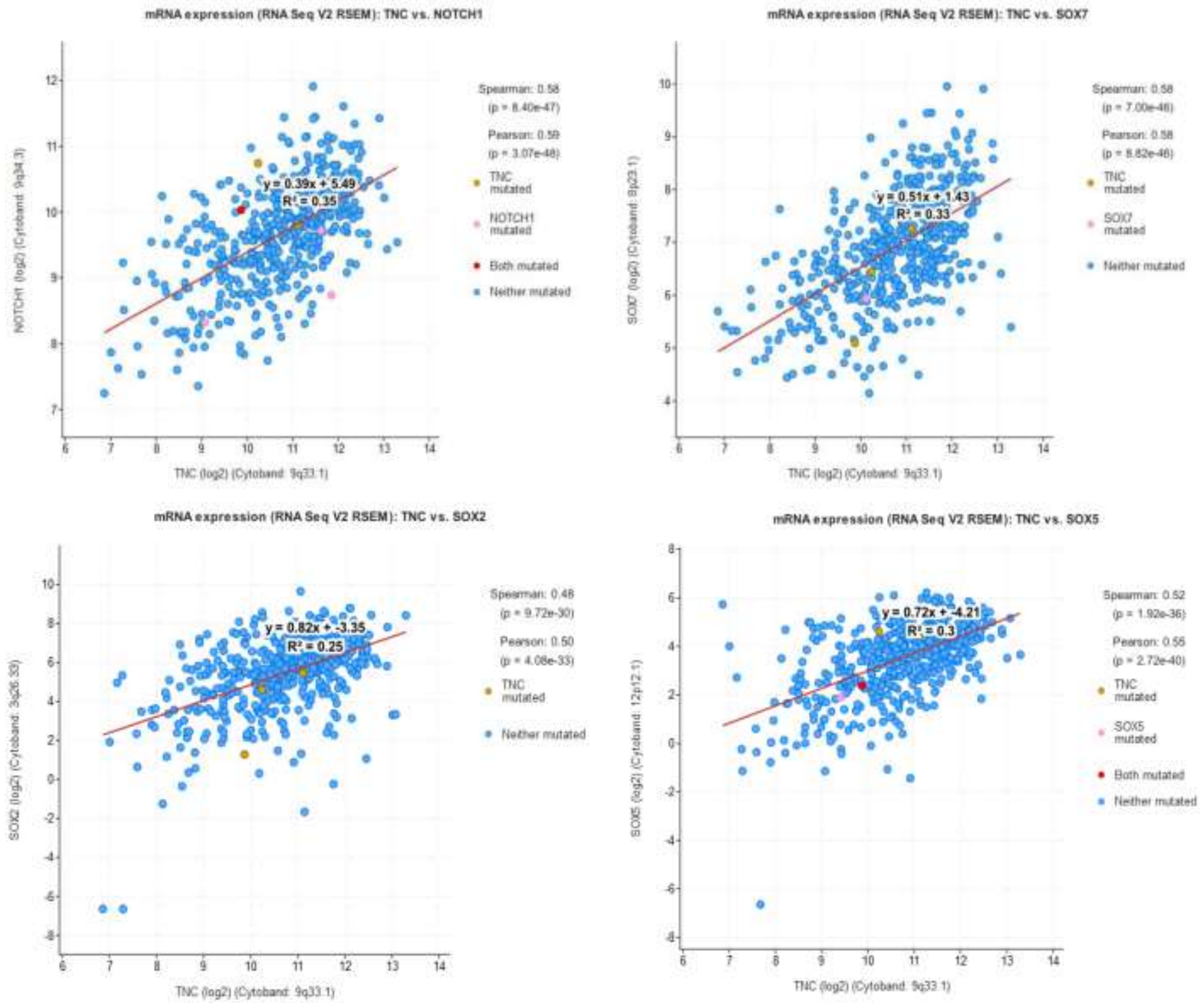

Supplementary Figure 3: Correlation analysis of TNC and de-differentiation markers

Supplementary Table 1: List of genes showing significant co-occurrence with TNC in NEPC dataset

Gene	Cytoband	Samples with alteration in TNC altered group	Samples with alteration in unaltered group	Log Ratio	p-Value	q-Value	Tendency
NOTCH1	9q34.3	22 (84.62%)	2 (2.47%)	5.1	9.71E-17	7.22E-15	Co-occurrence
NOTCH3	19p13.12	16 (61.54%)	4 (4.94%)	3.64	3.81E-09	2.99E-08	Co-occurrence
NOTCH2	1p12	16 (61.54%)	5 (6.17%)	3.32	1.43E-08	7.82E-08	Co-occurrence
NOTCH2NLA	1q21.1	17 (65.38%)	9 (11.11%)	2.56	1.65E-07	5.18E-07	Co-occurrence
NOTCH4	6p21.32	11 (42.31%)	5 (6.17%)	2.78	4.96E-05	7.29E-05	Co-occurrence
QSOX2	9q34.3	21 (80.77%)	2 (2.47%)	5.03	1.50E-15	6.79E-14	Co-occurrence
SOX9	17q24.3	19 (73.08%)	3 (3.70%)	4.3	1.47E-12	3.85E-11	Co-occurrence
SOX14	3q22.3	17 (65.38%)	6 (7.41%)	3.14	7.37E-09	4.57E-08	Co-occurrence
SOX2	3q26.33	17 (65.38%)	7 (8.64%)	2.92	2.27E-08	1.16E-07	Co-occurrence
SOX13	1q32.1	19 (73.08%)	12 (14.81%)	2.3	6.25E-08	2.39E-07	Co-occurrence
SOX3	Xq27.1	18 (69.23%)	10 (12.35%)	2.49	6.92E-08	2.54E-07	Co-occurrence
SOX30	5q33.3	15 (57.69%)	5 (6.17%)	3.22	8.66E-08	3.06E-07	Co-occurrence
SOX8	16p13.3	14 (53.85%)	4 (4.94%)	3.45	1.44E-07	4.66E-07	Co-occurrence
QSOX1	1q25.2	18 (69.23%)	11 (13.58%)	2.35	1.65E-07	5.18E-07	Co-occurrence
SOX12	20p13	15 (57.69%)	7 (8.64%)	2.74	7.40E-07	1.90E-06	Co-occurrence
SOX18	20q13.33	17 (65.38%)	11 (13.58%)	2.27	9.12E-07	2.22E-06	Co-occurrence
SOX4	6p22.3	12 (46.15%)	4 (4.94%)	3.22	3.92E-06	7.65E-06	Co-occurrence
SOX5	12p12.1	9 (34.62%)	3 (3.70%)	3.22	1.15E-04	1.61E-04	Co-occurrence
SOX6	11p15.2	8 (30.77%)	2 (2.47%)	3.64	1.51E-04	2.05E-04	Co-occurrence
SOX11	2p25.2	10 (38.46%)	6 (7.41%)	2.38	4.47E-04	5.75E-04	Co-occurrence
SOX10	22q13.1	6 (23.08%)	1 (1.23%)	4.22	7.40E-04	8.89E-04	Co-occurrence
SOX21	13q32.1	7 (26.92%)	3 (3.70%)	2.86	1.75E-03	2.05E-03	Co-occurrence
SOX17	8q11.23	15 (57.69%)	20 (24.69%)	1.22	2.37E-03	2.78E-03	Co-occurrence
SOX1	13q34	8 (30.77%)	6 (7.41%)	2.05	4.91E-03	5.52E-03	Co-occurrence
SOX7	8p23.1	6 (23.08%)	3 (3.70%)	2.64	6.11E-03	6.82E-03	Co-occurrence
SOX15	17p13.1	3 (11.54%)	0 (0.00%)	>10	0.0131	0.0142	Co-occurrence
WNT7A	3p25.1	20 (76.92%)	4 (4.94%)	3.96	7.76E-13	2.36E-11	Co-occurrence
WNT5A	3p14.3	20 (76.92%)	5 (6.17%)	3.64	3.61E-12	9.21E-11	Co-occurrence
WNT3	17q21.31-q21.32	18 (69.23%)	3 (3.70%)	4.22	1.37E-11	2.81E-10	Co-occurrence
WNT9B	17q21.32	18 (69.23%)	3 (3.70%)	4.22	1.37E-11	2.81E-10	Co-occurrence
WNT3A	1q42.13	18 (69.23%)	6 (7.41%)	3.22	1.05E-09	1.14E-08	Co-occurrence
WNT9A	1q42.13	18 (69.23%)	6 (7.41%)	3.22	1.05E-09	1.14E-08	Co-occurrence
WNT1	12q13.12	13 (50.00%)	1 (1.23%)	5.34	7.01E-09	4.57E-08	Co-occurrence
WNT10B	12q13.12	13 (50.00%)	1 (1.23%)	5.34	7.01E-09	4.57E-08	Co-occurrence
WNT11	11q13.5	17 (65.38%)	6 (7.41%)	3.14	7.37E-09	4.57E-08	Co-occurrence
WNT8A	5q31.2	13 (50.00%)	3 (3.70%)	3.75	2.12E-07	6.31E-07	Co-occurrence
WNT2B	1p13.2	10 (38.46%)	2 (2.47%)	3.96	7.20E-06	1.29E-05	Co-occurrence
WNT8B	10q24.31	9 (34.62%)	2 (2.47%)	3.81	3.41E-05	5.26E-05	Co-occurrence
WNT2	7q31.2	12 (46.15%)	8 (9.88%)	2.22	1.46E-04	2.02E-04	Co-occurrence
WNT4	1p36.12	5 (19.23%)	0 (0.00%)	>10	6.19E-04	7.75E-04	Co-occurrence
WNT10A	2q35	7 (26.92%)	2 (2.47%)	3.45	6.31E-04	7.76E-04	Co-occurrence
WNT6	2q35	7 (26.92%)	2 (2.47%)	3.45	6.31E-04	7.76E-04	Co-occurrence

WNT5B	12p13.33	9 (34.62%)	5 (6.17%)	2.49	7.37E-04	8.89E-04	Co-occurrence
WNT16	7q31.31	11 (42.31%)	11 (13.58%)	1.64	2.97E-03	3.40E-03	Co-occurrence
WNT7B	22q13.31	3 (11.54%)	0 (0.00%)	>10	0.0131	0.0142	Co-occurrence
FZD6	8q22.3	24 (92.31%)	20 (24.69%)	1.9	6.73E-10	8.59E-09	Co-occurrence
FZD2	17q21.31	15 (57.69%)	3 (3.70%)	3.96	5.83E-09	4.32E-08	Co-occurrence
FZD1	7q21.13	16 (61.54%)	6 (7.41%)	3.05	4.66E-08	1.81E-07	Co-occurrence
FZD4	11q14.2	15 (57.69%)	7 (8.64%)	2.74	7.40E-07	1.90E-06	Co-occurrence
FZD9	7q11.23	14 (53.85%)	7 (8.64%)	2.64	3.66E-06	7.34E-06	Co-occurrence
FZD5	2q33.3	9 (34.62%)	2 (2.47%)	3.81	3.41E-05	5.26E-05	Co-occurrence
FZD7	2q33.1	9 (34.62%)	2 (2.47%)	3.81	3.41E-05	5.26E-05	Co-occurrence
FZD8	10p11.21	7 (26.92%)	1 (1.23%)	4.45	1.68E-04	2.24E-04	Co-occurrence
FZD10	12q24.33	7 (26.92%)	5 (6.17%)	2.12	7.96E-03	8.87E-03	Co-occurrence
MSI2	17q22	18 (69.23%)	4 (4.94%)	3.81	6.86E-11	1.21E-09	Co-occurrence
MSI1	12q24.31	10 (38.46%)	8 (9.88%)	1.96	1.73E-03	2.05E-03	Co-occurrence
NANOG	12p13.31	9 (34.62%)	2 (2.47%)	3.81	3.41E-05	5.26E-05	Co-occurrence
NANOGNB	12p13.31	9 (34.62%)	2 (2.47%)	3.81	3.41E-05	5.26E-05	Co-occurrence
LRG1	19p13.3	14 (53.85%)	1 (1.23%)	5.45	1.05E-09	1.14E-08	Co-occurrence
LRGUK	7q33	13 (50.00%)	8 (9.88%)	2.34	3.74E-05	5.61E-05	Co-occurrence
KLRG1	12p13.31	8 (30.77%)	2 (2.47%)	3.64	1.51E-04	2.05E-04	Co-occurrence
KLRG2	7q34	13 (50.00%)	10 (12.35%)	2.02	1.56E-04	2.11E-04	Co-occurrence
PLRG1	4q31.3	9 (34.62%)	4 (4.94%)	2.81	3.13E-04	4.06E-04	Co-occurrence
